# Effects of verification stage intensity and duration on $\dot{V}O_{{2\max }}$ determination

**DOI:** 10.1007/s00421-026-06314-6

**Published:** 2026-06-29

**Authors:** Andrew Foster, Erin J. Dennis, Stephanie P. Kurti, Nicholas D. Luden, Michael J. Saunders, Christopher J. Womack

**Affiliations:** Human Performance Laboratory, Department of Kinesiology, James Madison University, Harrisonburg, VA 22807 USA

**Keywords:** Cardiorespiratory fitness, Exercise testing, Graded exercise test, Maximal oxygen uptake, Verification stages

## Abstract

**Purpose:**

Verification stages are recommended for maximal oxygen consumption (V̇O_₂max_) determination, but there is currently no standardization of verification stage design. We sought to determine if the length of recovery and the initial intensity of subsequent exercise influence the effectiveness of verification stages during testing for V̇O_₂max_.

**Methods:**

Twenty-seven participants (20 males, 7 females) completed four graded exercise tests (GXT). Each session included an identical incremental V̇O_₂max_ (iV̇O_₂max_) protocol, followed by one of four verification stages with combinations of short (5 min) and long (15 min) rest periods, and submaximal (one stage below maximal workload) and supramaximal (one stage above maximal workload) starting intensities.

**Results:**

Although iV̇O_₂max_ and verification V̇O_₂max_ (vV̇O_₂max_) were similar across all protocols, 11–19% of participants achieved higher values during verification. About 24% of individuals who did not exhibit a V̇O_₂_ plateau during the GXT exhibited a vV̇O_₂max_ > iV̇O_₂max_ in the submaximal verification protocols compared to 6–14% for the supramaximal protocols. Conclusions: Verification stages using initial submaximal intensities may increase the likelihood of achieving V̇O_₂max_.

## Introduction

Maximal oxygen uptake (V̇O_2max_) is one of the most tested variables in exercise science and is associated with numerous long-term health outcomes (Wei et al. 1999). Evidence of a plateau in V̇O_2_ at the end of a graded exercise test (GXT) has historically been used to ensure V̇O_2max_ was reached. However, plateaus are often not evident, as plateau incidence rates range from 20 – 60% if appropriate criteria are used (Niemeyer et al. 2021). Furthermore, although traditional secondary criteria such as achievement of a particular heart rate or respiratory exchange ratio (RER) have been updated to reduce misclassification of V̇O_2max_ attainment (Wagner et al. 2020; Knaier et al. 2019), it is not known if use of a verification stage enhances V̇O_2max_ determination beyond using a plateau and secondary criteria.

Verification stages have been suggested as a method to ensure achievement of V̇O_2max_. A verification stage is conducted after a recovery period following the GXT and begins at a workload close to the maximal workload from the GXT. Several studies have found that verification stages result in similar V̇O_2max_ values as achieved during the GXT (Midgley et al. 2006; Foster et al. 2007; Kirkeberg et al. 2011; Costa et al. 2021). Conversely, Bhammar et al. (2017) reported that verification stages result in a significantly higher V̇O_2max_ compared to the V̇O_2max_ achieved during the GXT. Finally, verification stages yield higher V̇O_2max_ values in about 31% of tests (Kontos et al. 2023). This suggests that a verification stage will either confirm V̇O_2max_ was reached during the GXT or allow for the measurement of a higher V̇O_2max_.

The duration of the recovery period has varied in the literature, and researchers have suggested that recovery stage duration does not impact verification stage efficacy. Nolan et al. (2014) found no difference between 20 and 60 min recovery stages, which may suggest that rest period duration is not an important factor. However, recommended rest period duration is 5–15 min (Schaun 2017), and no known studies have addressed whether the lower and higher ends of this recommended range influence verification stage efficacy.

There is also significant variability in the intensity selected for the verification stage, ranging from lower than the last stage completed during the GXT to workloads higher than the highest achieved from the GXT. Iannetta et al. (2020) showed that workloads at 110% of peak GXT workload can reduce the ability to achieve V̇O_2max_, while Nolan et al. (2014) showed that a verification stage intensity of 105% of peak GXT workload was superior to 115%. Recent work has suggested that verification testing may add limited value, regardless of whether fixed submaximal or supramaximal intensities are used (Bruggisser et al. 2024). However, verification stages that progressively increase in intensity may represent a distinct approach that could influence both physiological responses and tolerance to the test. Additionally, the interaction between recovery duration and verification-stage structure, particularly when using a progressive verification-stage design, has not been well characterized.

Therefore, the purposes of this study were to determine if the length of the recovery stage and/or the intensity of the verification stage had an impact on verification stage efficacy. We hypothesized that the intensity and duration of the verification stage would not influence the effectiveness of the verification stage.

## Methods

### Participants

This study included 27 participants (20 males and 7 females) recruited from the campus community. The sample size is comparable to prior repeated-measures designs examining verification stage protocols (Kontos et al. 2023; Nolan et al. 2014). All participants were free from known cardiovascular, metabolic, and/or renal disease and free from signs/symptoms of those same diseases. Average age was 20.9 ± 1.4 y, average height was 176.6 ± 7.4 cm, and average body mass was 75.5 ± 13.5 kg. Participants were not allowed to consume any food, drink, or caffeine 3 h prior to the exercise test, except water. Participants also refrained from exercise on the day of testing. Written informed consent was obtained from all participants prior to participation and that the protocol was approved by the James Madison University Institutional Review Board (IRB #20-1572). This study was implemented in accordance with the Declaration of Helsinki.

### GXT

Each participant completed four separate V̇O_2max_ tests separated by at least 48 h. For each initial graded exercise test, starting speed was 4.8 km/h and increased by 0.8 km/h every minute until 9.7 km/h was reached. After this point, treadmill grade was increased by 3.0% every minute until volitional exhaustion. This protocol was selected as it tends to elicit fatigue in younger, college-aged participants in around 12 min (Paton et al. 2004). Oxygen uptake (V̇O_2_) was continuously measured breath x breath using a Parvomedics metabolic measurement system (Parvomedics; Salt Lake City, UT). A Polar T31 heart rate monitor (Polar Electro, Inc.; Bethpage, NY) was used to obtain heart rate at the end of each minute. Rating of perceived exertion (RPE) were obtained every minute using a Borg 6–20 RPE scale. Verbal encouragement was provided to the participants during the test. The highest 30 s average achieved during the GXT was defined as V̇O_2max_ for the incremental test (iV̇O_2max_). Maximal workload was defined as the highest stage achieved if the subject completed at least 30 s of that stage. A V̇O₂ plateau at the end of the GXT was defined as an increase in relative V̇O_₂_ of less than 1.69 mL·kg⁻1 min⁻1 between the final two stages, as this value falls below the 95% confidence interval for the expected increase in V̇O_₂_ per stage for this GXT protocol (Kontos et al. 2023).

### Verification stage

After each GXT, an active rest period consisting of a walk at 1.5–1.7 mph occurred, followed by a verification stage. The duration of the rest period (5 or 15 min) and the starting intensity of the verification stage differed across the four trials. Verification stages began at either a submaximal (one stage below the maximal workload achieved during the GXT) and supramaximal (one stage above maximal workload achieved during the GXT) workload. Importantly, all verification stages were performed as progressive tests, with intensity increasing each minute in the same manner as the GXT until volitional exhaustion. Verbal encouragement was provided during the verification stage.

The four different trials were: 1) short duration/submaximal, 2) short duration/supramaximal, 3) long duration/submaximal, 4) long duration/supramaximal. Trial order was counterbalanced (1-2-3-4, 2-3-4-1, 3-4-1-2, 4-1-2-3), with seven participants completed orders 1–3, and six completing order 4. V̇O_2_ and HR were measured throughout the verification stages as described for the GXT. The highest 30 s average for V̇O_2_ was defined as verification stage V̇O_2max_ (vV̇O_2max_).

### Statistical analysis

All statistical analyses were performed using SPSS statistical software. V̇O_2max_ was compared between trials using a repeated measures analysis of variance (ANOVA) with exercise phase (GXT, verification stage) and protocol (short/submaximal, long/submaximal, short/supramaximal, and long/supramaximal) as within-subject factors. Duration of the GXT, duration of the verification stage, RER_max_, HR_max_ and RPE_max_ from the GXT were compared across protocols using a repeated measures ANOVA. Post-hoc comparisons were made using paired t-tests with a Bonferroni correction. The coefficient of variation for iV̇O_2max_ across the four testing sessions was 4%. Thus, vV̇O_2max_ was required to exceed iV̇O_2max_ by more than 4% to be considered meaningfully higher or lower, respectively. Statistical significance was accepted a priori at an alpha level of < 0.05.

## Results

Mean V̇O_2max_ was similar between iV̇O_2max_ and vV̇O_2max_ across all protocols (Table 1).

**Table 1:** Average (± SD) absolute and relative iV̇O_2max_ and vV̇O_2max_ along with maximal heart rate for all four protocols

Protocol	iV̇O_₂max_ (L min⁻^1^)	vV̇O_₂max_ (L min⁻^1^)	iV̇O_₂max_ (mL kg⁻^1^ min⁻^1^)	vV̇O_₂max_ (mL kg⁻^1^ min⁻^1^)	GXT HRmax (bpm)
Short/Submaximal	3.79 ± 0.89	3.81 ± 0.93	50.32 ± 9.42	50.47 ± 10.00	188 ± 10
Short/Supramaximal	3.82 ± 0.85	3.73 ± 0.89	50.89 ± 8.63	49.66 ± 9.61	188 ± 9
Long/Submaximal	3.81 ± 0.91	3.77 ± 0.91	50.56 ± 9.20	50.06 ± 9.53	188 ± 10
Long/Supramaximal	3.76 ± 0.93	3.73 ± 0.89	49.91 ± 9.80	49.52 ± 9.42	187 ± 11

**Fig. 1:**
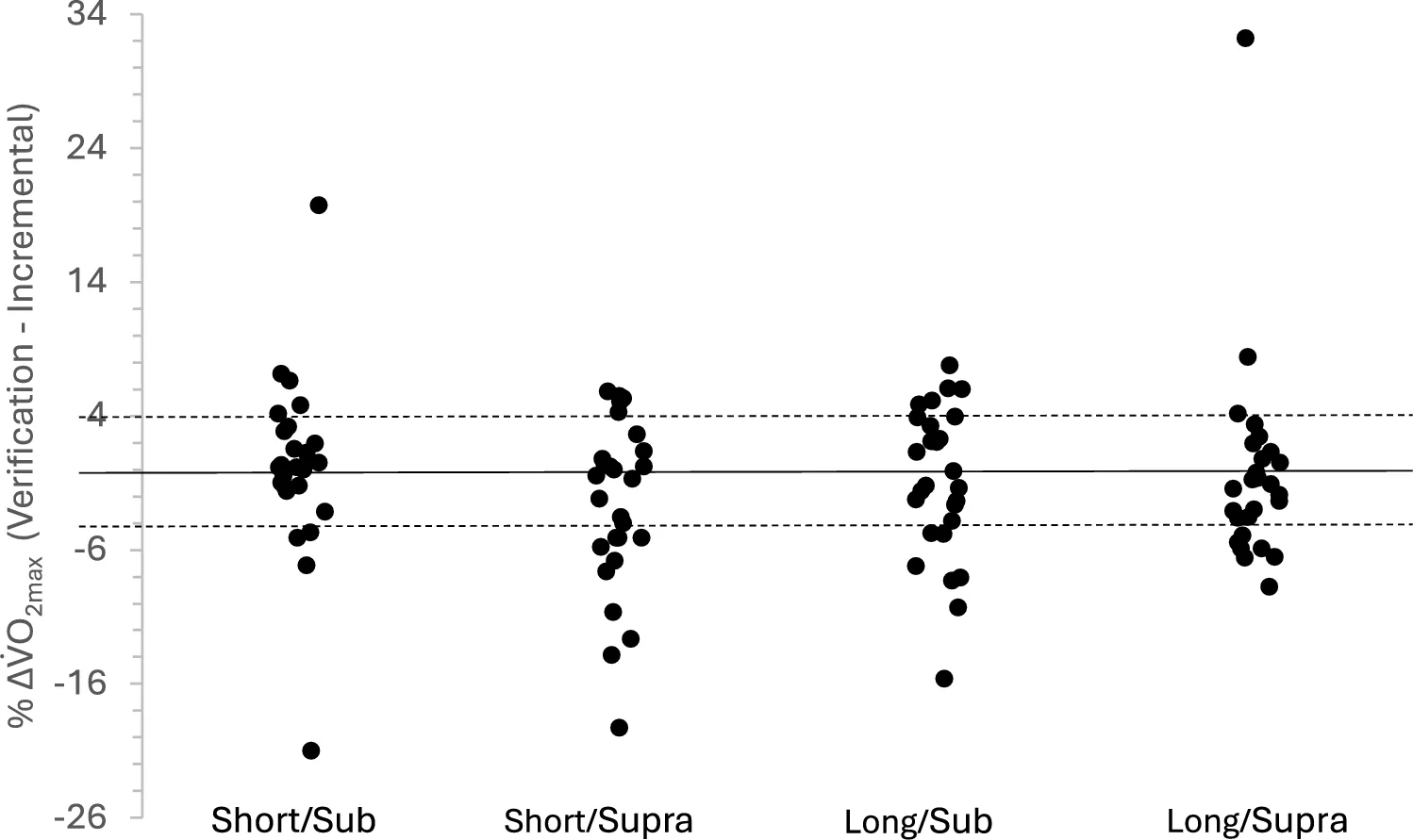
Percent difference between verification stage V̇O_2max_ and incremental test V̇O_2max_ for all four protocols. The solid line depicts zero difference and the dashed lines depict the upper and lower limits (4%) that were used to classify verification-stage V̇O_₂max_ as higher, similar, or lower than incremental-test V̇O_₂max_

RER_max_ (range = 1.14 ± 0.11 to 1.17 ± 0.10), HR_max_ (range = 187 ± 11 to 188 ± 10 beats/min), and RPE_max_ (range = 18.4 ± 1.8 to 18.8 ± 1.3) were not significantly different across the four protocols (p > 0.05). The individual data for the percent difference between iV̇O_2max_ and vV̇O_2max_ is shown in Fig. 1.

The proportion of tests with vV̇O_2max_ higher, equal to, and lower than the iV̇O_2max_ varied (Table 2). The short/submaximal protocol had the lowest proportion of tests in which vV̇O_2max_ was less than iV̇O_2max_. Furthermore, the proportion of tests where iV̇O_2max_ = vV̇O_2max_ was higher in the short/submaximal than in the long/submaximal. Verification-stage outcomes stratified by the presence or absence of a V̇O_₂_ plateau are shown in Table 3. Among participants who did not exhibit a V̇O_₂_ plateau during the incremental test, 3 of 14, 1 of 17, 4 of 19, and 3 of 22 participants achieved a vV̇O_₂max_ that exceeded iV̇O_2max_ by more than 4% for the short/submaximal, short/supramaximal, long/submaximal, and long/supramaximal conditions, respectively. Furthermore, vV̇O_2max_ was equal to or greater than iV̇O_2max_ in 12 of 14, 14 of 17, 15 of 19, and 17 of 22 in participants who did not demonstrate a plateau.

**Table 2:** The number of participants with verification stage V̇O_2max_ 4% over, within 4%, and 4% under the incremental stage V̇O_2max_

	Short/Submaximal	Short/Supramaximal	Long/Submaximal	Long/Supramaximal
Higher	5 (18.5%)	5 (18.5%)	5 (18.5%)	3 (11.1%)
Same	18 (66.7%)	12 (44.4%)	15 (55.6%)	17 (63.0%)
Lower	4 (14.8%)	10 (37.0%)	6 (22.2%)	7 (25.9%)

**Table 3:** Verification-stage outcomes stratified by presence or absence of a V̇O₂ plateau during the incremental test

Protocol	Plateau (n)	Higher	Same	Lower
Short/Submaximal	13	2 (15.4)	9 (69.2)	2 (15.4)
Short/Supramaximal	10	4 (40.0)	5 (50.0)	1 (10.0)
Long/Submaximal	8	1 (12.5)	5 (62.5)	2 (25.0)
Long/Supramaximal	5	0 (0.0)	4 (80.0)	1 (20.0)

Protocol	No Plateau (n)	Higher	Same	Lower
Short/Submaximal	14	3 (21.4)	9 (57.1)	2 (21.4)
Short/Supramaximal	17	1 (5.9)	13 (76.5)	3 (17.6)
Long/Submaximal	19	4 (21.1)	11 (57.9)	4 (21.1)
Long/Supramaximal	22	3 (13.6)	14 (63.6)	5 (22.7)

Values are presented as n (percentage)

Among participants who did not exhibit a V̇O₂ plateau and whose verification-stage V̇O_₂max_ was equal to or greater than the incremental test, 10 of 12, 9 of 10, 11 of 17, and 15 of 17 participants met the HR criterion for maximal effort (≥ 93% age-predicted maximal heart rate using the Tanaka (2001) equation) for the short/submaximal, short/supramaximal, long/submaximal, and long/supramaximal conditions, respectively. Similarly, 8 of 12, 7 of 10, 9 of 17, and 10 of 17 participants met the RER criterion (RER ≥ 1.13). The secondary criteria thresholds were based on those suggested for this age group by Wagner et al. (2020).

Exercise test duration data are shown in Table 4. There were no significant differences for GXT duration across the protocols. The supramaximal verification stages were significantly (p < 0.05) shorter than the submaximal verification stages and the short/supramaximal verification stage was significantly (p < 0.05) shorter than all protocols.

**Table 4:** Duration of graded exercise test and verification stages across experimental conditions

Protocol	GXT duration (min)	Verification duration (min)
Short/Submaximal	11.52 ± 1.48 (9.33 – 14.50)	1.98 ± 0.39 (1.22 – 2.67)
Short/Supramaximal	11.44 ± 1.48 (9.00 – 14.08)	1.28 ± 0.31^1,3,4^ (1.00 – 1.93)
Long/Submaximal	11.34 ± 1.64 (9.17 – 14.33)	2.19 ± 0.51 (1.08 – 3.00)
Long/Supramaximal	11.28 ± 1.56 (8.33 – 14.33)	1.54 ± 0.35^1,2,3^ (1.17 – 2.88)

Values are mean ± SD (range). ^1^Significantly different than Short/Submaximal, ^2^Significantly different than Short/Supramaximal, ^3^Significantly different than Long/Submaximal, ^4^Significantly different than Long/Supramaximal

## Discussion

There was no significant difference between average iV̇O_2max_ and vV̇O_2max_ across the different protocols, regardless of rest period duration and starting intensity. This is consistent with Costa et al. (2021), who reported that GXT V̇O_2max_ will yield similar results to verification stage V̇O_2max_, regardless of potential moderators, such as verification phase intensity, verification phase duration, or type of recovery. When observing the individual data, the short/submaximal protocol appears to be least likely to produce a vV̇O_2max_ lower than iV̇O_2max_. Overall, the short/submaximal protocol yielded vV̇O_2max_ values that were the same or higher than iV̇O_2max_ in 85.2% of tests compared to the short/supramaximal (62.9%), long/submaximal (74.1%), and long/supramaximal (74.1%) protocols. Previous research has suggested that excessively high workloads can reduce the ability to achieve V̇O_2max_ (Nolan et al. 2014; Iannetta et al. 2020). Similar to the present study, Nolan et al. (2014) compared several different verification stages including rest durations of 20 vs. 60 min and intensities of 105% and 115% of GXT maximal workload. The verification stages performed at 115% of maximal workload appeared to have a higher portion of participants achieving a vV̇O_2max_ less than the iV̇O_2max_ (Nolan et al. 2014). Thus, it is possible that higher intensity verification stages could limit verification stage efficacy due to participants reaching premature fatigue before V̇O_2max_ is achieved. As evidence of this, beginning the verification stages at a supramaximal workload resulted in a shorter verification stage duration in the present study.

The primary utility of a verification stage is to confirm V̇O_₂max_ achieved during the GXT or, in some cases, to provide an opportunity to obtain a higher value in individuals who do not exhibit a plateau. Among participants who did not exhibit a V̇O₂ plateau during the incremental test, approximately 21% achieved a higher V̇O_₂max_ during verification in the submaximal protocols, compared to 6–14% in the supramaximal protocols. This suggests that incremental verification stages that start at a submaximal intensity may be preferable. However, the relatively small proportion of such cases also suggests that verification testing may not be necessary in all individuals. The utility of the verification stage may depend on investigator preference and the relative need for fidelity in this measure. Out of the 108 tests in the present study, 43 did not plateau but did achieve both secondary criteria. In 35 of these tests (81%), the vV̇O_₂max_ was the same or greater than iV̇O_₂max_ while vV̇O_₂max_ exceeded iV̇O_₂max_ in only 5 (11%) of the tests. It is reasonable to conclude from this that using valid plateau and secondary criteria would likely result in successful V̇O_₂max_ determination in most tests. However, in certain research scenarios, the addition of the verification stage may be worth the extra effort on the part of the participant and investigator. A practical suggestion is to only utilize the verification stage if neither the plateau nor secondary criteria are evident thus potentially eliminating extra effort by the participants.

A shorter rest period may be more likely to produce a higher V̇O_2max_ due to V̇O_2_ remaining somewhat elevated following the short rest period, whereas longer rest periods may allow V̇O_2_ to return closer to resting values. In the present study, the vV̇O_2max_ was lower than the iV̇O_2max_ value for both long rest duration protocols 22–26% of the time. This was also the case for the short/supramaximal (37%), but not the short/submaximal protocol, where the proportion of tests with a lower vV̇O_2max_ dropped to 14.8%.

A recommendation could be made based on the current data to employ verification stages similar to the short/submaximal stage used in the present study. In addition to the greatest proportion of tests above or equal to iV̇O_2max_, this protocol was among the best at eliciting a higher vV̇O_2max_ in individuals not exhibiting a plateau. Furthermore, less time would be taken up with the recovery stage, and the use of a starting point below maximal workload could be beneficial for subject comfort. Some limitations to this study were that our findings are limited to treadmill exercise using our specific protocol. It is unknown if these findings can be generalized to other testing modalities or to other treadmill protocols with different workload increments. Despite using plateau criteria based on V̇O_2_ responses to this exact protocol in our laboratory, there were some subjects who exhibited an elevated vV̇O_2max_ despite meeting the threshold criteria for a plateau. Thus, it is possible that daily variability of physiological responses and metabolic measurements resulted in tests where a true plateau was not achieved but met our criteria. Similarly, although the 4% elevation was required for vV̇O_2max_ to exceed iV̇O_2max_, it is possible that some tests reached the upper range of biological and measurement variability. Furthermore, the participants consisted of young, relatively fit individuals, so the relative efficacy of the verification stages that were tested may not translate to older and/or sedentary populations. Finally, the sample used in the present study consisted disproportionally of males. This was not intended and was entirely due to differing response rates to advertising for the study. It is unknown whether the findings of the present study extend to males and females equally.

When verification stages are employed, practitioners may want to utilize shorter rest periods and submaximal intensities to reduce testing time and maximize the likelihood of obtaining higher vV̇O_2max_ values during verification.

## Data Availability

The datasets generated and analyzed during the current study are available from the corresponding author on reasonable request.

## Abbreviation

ANOVA: Analysis of variance
GXT: Graded exercise test
HR: Heart rate
HR_max_: Maximal heart rate
iV̇O_2max_: Maximal oxygen uptake from the incremental test
RER: Respiratory exchange ratio
RPE: Rating of perceived exertion
V̇O_2_: Oxygen uptake
V̇O_2max_: Maximal oxygen uptake
vV̇O_2max_: Maximal oxygen uptake from the verification stage
